# Ultra-conserved sequences in the genomes of highly diverse *Anopheles* mosquitoes, with implications for malaria vector control

**DOI:** 10.1093/g3journal/jkab086

**Published:** 2021-03-17

**Authors:** Samantha M O’Loughlin, Annie J Forster, Silke Fuchs, Tania Dottorini, Tony Nolan, Andrea Crisanti, Austin Burt

**Affiliations:** 1 Department of Life Sciences, Imperial College London, Ascot SL5 7PY, UK; 2 School of Veterinary Medicine and Science, Sutton Bonington Campus, University of Nottingham, Leicestershire LE12 5RD, UK; 3 Liverpool School of Tropical Medicine, Liverpool L3 5QA, UK

**Keywords:** Anopheles, gene drive, conserved, malaria

## Abstract

DNA sequences that are exactly conserved over long evolutionary time scales have been observed in a variety of taxa. Such sequences are likely under strong functional constraint and they have been useful in the field of comparative genomics for identifying genome regions with regulatory function. A potential new application for these ultra-conserved elements (UCEs) has emerged in the development of gene drives to control mosquito populations. Many gene drives work by recognizing and inserting at a specific target sequence in the genome, often imposing a reproductive load as a consequence. They can therefore select for target sequence variants that provide resistance to the drive. Focusing on highly conserved, highly constrained sequences lowers the probability that variant, gene drive-resistant alleles can be tolerated. Here, we search for conserved sequences of 18 bp and over in an alignment of 21 *Anopheles* genomes, spanning an evolutionary timescale of 100 million years, and characterize the resulting sequences according to their location and function. Over 8000 UCEs were found across the alignment, with a maximum length of 164 bp. Length-corrected gene ontology analysis revealed that genes containing *Anopheles* UCEs were over-represented in categories with structural or nucleotide-binding functions. Known insect transcription factor binding sites were found in 48% of intergenic *Anopheles* UCEs. When we looked at the genome sequences of 1142 wild-caught mosquitoes, we found that 15% of the *Anopheles* UCEs contained no polymorphisms. Our list of *Anopheles* UCEs should provide a valuable starting point for the selection and testing of new targets for gene-drive modification in the mosquitoes that transmit malaria.

## Introduction

DNA sequences that are highly conserved over long evolutionary timescales have been identified in many organisms. Some of these sequences show complete conservation at the nucleotide level and are often known as ultra-conserved elements (UCEs). Originally, UCEs were defined as sequences of at least 200 bp that were identical between human, mouse, and rat genomes (Bejerano *et al.* 2004). Subsequently, the search for UCEs has been extended to other vertebrates, insects, and plants (*e.g.*Siepel *et al.* 2005; Baxter *et al.* 2012; Makunin *et al.* 2013; Quattrini *et al.* 2018), and to sequences of length 50 bp or more.

There are several reasons why UCEs are of interest. First, in the field of comparative genomics, UCEs are thought to represent functionally important regions. While there is still some mystery around why sequences might be conserved at the nucleotide level over long evolutionary timescales, it has been shown that UCEs: (1) often are involved in the regulation of transcription of genes, especially essential genes involved in development (*e.g.* (Visel *et al.* 2008); (2) may have a role in chromosomal structure (*e.g.*Chiang *et al.* 2008); and (3) are sometimes non-coding RNA genes (*e.g.*Kern *et al.* 2015). Even UCEs in protein-coding regions may have multi-functional roles (Warnefors *et al.* 2016). Second, UCEs can act as probes to facilitate genomic sequencing of non-model organisms using sequence-capture methods (Faircloth *et al.* 2012). Third, alterations in UCEs have been shown to have an association with human cancers (*e.g.*Calin *et al.* 2007; Lin *et al.* 2012).

A new potential role for UCEs has recently emerged in the fight against malaria using gene drive mosquitoes (Kyrou *et al.* 2018). *Anopheles* mosquitoes are the vectors of malaria parasites, and mosquito control has been responsible for much of the recent success in the reduction of malaria cases [78% of the 663 million malaria cases averted globally since 2000 (Bhatt *et al.* 2015)]. Progress in reducing malaria cases has stalled (WHO 2018), probably in part due to resistance of the mosquitoes against commonly used pesticides. One novel method under consideration is the development of mosquitoes containing gene drives that either reduce the population size (Windbichler *et al.* 2011; Hammond *et al.* 2016) or make them unable to transmit the malaria parasite (Gantz *et al.* 2015). Both methods currently rely on nuclease-based synthetic gene drive systems that introduce a desired trait at a precise genomic location, spreading it in a target population at such a rate that outweighs fitness costs associated with the trait (Burt 2003). The technologies include RNA-guided endonucleases (such as CRISPR/Cas9) and homing endonucleases (Windbichler *et al.* 2011; Jinek *et al.* 2012). These enzymes recognize and cleave a particular target size of about 18 bp. When the sequence coding for these enzymes is engineered into its own target site in the genome and is expressed in the germline, it creates a double-strand break in the homologous chromosome. The break will usually be repaired by homology-directed repair using the drive-containing chromosome as a template which results in conversion of the repaired chromosome to also contain the drive element in greater than the usual 50% inheritance rate among the gametes. An efficient gene drive can be inherited by almost 100% of progeny (Hammond *et al.* 2016). Theoretical and laboratory studies have shown that changes to the recognition site can result in alleles that cannot be recognized or cleaved. If these alleles confer increased fitness compared with the wild-type allele in the presence of the gene drive they can be expected to spread and retard the spread of the gene drive (Deredec *et al.* 2008; Hammond *et al.* 2017; Unckless *et al.* 2017). For population suppression gene drives that are designed to impair essential genes, the selection pressure for resistance alleles to arise is high. These alleles can arise from standing variation at the target site in a wild population or may come about from the action of the endonuclease. This is because non-homologous end joining can sometimes repair the double-strand break, and random insertions and deletions can be introduced to the target site.

Two of the most important vector species in sub-Saharan Africa are the close relatives *Anopheles gambiae* and *Anopheles coluzzii*, both of which are highly genetically diverse. A study of 765 mosquitoes in phase 1 of Ag1000G project, which looked to sample genetic diversity among these two species in the wild, through the resequencing of wild-caught individuals across Africa (Anopheles gambiae 1000 Genomes Consortium *et al.* 2017), found a polymorphism on average every 2.2 bases of the accessible genome. Nucleotide diversity (π) ranged from ∼0.008 to ∼0.015 per population sampled, and even non-degenerate sites (which are expected to be strongly constrained) had an average π of ∼0.0025.

Proof of principle for retarding the evolution of resistance to nuclease-based gene drive by targeting an evolutionarily conserved sequence has recently been demonstrated. A strain of mosquitoes with a CRISPR/Cas9 gene-drive targeting the *doublesex* gene fully suppressed laboratory caged populations of *An. gambiae* (Kyrou *et al.* 2018) without selecting for resistance. The CRISPR/Cas9 target sequence in this strain is an intron/exon junction that is highly conserved across the *An. gambiae* species complex, and only one rare single nucleotide polymorphism was found in the sequence in *An. gambiae* and *An. coluzzii* in the Ag1000G data. Consistent with the target site being a region of high functional constraint, monitoring of potential resistant mutations during the cage experiment revealed that although some indels had been introduced by the endonuclease, none of them showed signs of positive selection.

This strong constraint at the nucleotide level may exist at other loci in *An. gambiae*. The Ag1000G project looked for conserved putative CRISPR/Cas9 target sites (18 invariant bases followed by the -NGG motif necessary for Cas9 cleavage) in the 765 mosquitoes of Phase 1 of the project, and found 5474 genes containing such sequences. However, they note that more variation is likely to be found with further sampling.

Here, we take an approach that is likely to be more stringent in identifying functionally constrained sequences by searching for regions that are ultra-conserved across the whole *Anopheles* genus, which has a most recent common ancestor ∼100 million years ago (Neafsey *et al.* 2015). Although sequence constraint across such a long-time scale is not necessary for a good target (as indicated by the *doublesex* locus, which is ultra-conserved within the *An. gambiae* species complex, but shows less conservation outside the complex), we are hypothesizing that such highly conserved sequences will contain few polymorphisms in the wild *Anopheles gambiae* population, and any polymorphisms that do arise (either spontaneously or due to the action of the endonuclease) are likely to have strong fitness costs. We also do not confine our analysis to sequences compatible with any single nuclease architecture (*e.g.* the 5′-NGG-3′ PAM sequence required by the SpCas9 nuclease) since the range and flexibility of nuclease architectures is constantly expanding, meaning that these requirements may be relaxed (Anders *et al.* 2016; Chatterjee *et al.* 2018; Hu *et al.* 2018). We extracted UCEs from an alignment of the genomes of 21 *Anopheles* species and strains that were constructed by the *Anopheles* 16 genomes consortium (Neafsey *et al.* 2015). We used data from *Drosophila* orthologues to group genic UCEs according to potential phenotype. We then use the Ag1000G data (1142 *An. gambiae* and *An. coluzzii*) to see whether these conserved elements contain any variation in natural populations of potential target mosquito species.

The main aim of our study was to identify potential targets for vector control, but as these are the first UCEs to be identified from an alignment of the *Anopheles* genus, we also characterized the UCEs according to their locations in the genome, and performed functional classification analyses to see how they compare with UCEs identified in other taxa.

## Materials and methods

### Data

Two sources of genomic data were used in this study: a multi-species alignment file (MAF) from the *Anopheles* 16 genomes project (Neafsey *et al.* 2015) and variation data from phase 2 of the MalariaGEN *An. gambiae* 1000 genomes project (*Anopheles gambiae* 1000 Genomes Consortium *et al.* 2017). The *Anopheles* 16 genomes project multi-species alignment contains reference genomes from 21 *Anopheles* species and strains: *An. gambiae PEST, An. gambiae s.s., An. coluzzii, An. merus, An. arabiensis, An. quadriannulatus, An. melas, An. christyi, An. epiroticus, An. minimus, An. culicifaces, An. funestus, An. stephensi S1, An. stephensi I2, An. maculatus, An. farauti, An. dirus, An. sinensis, An. atroparvus, An. darlingi*, and *An. albimanus.* A description of the methods used to create the alignment is found in Neafsey *et al.* (2015). Phase 2 of the Ag1000G project comprises 1142 *An. gambiae*, *An. coluzzii* and hybrids, collected from 13 countries in Africa (The *Anopheles gambiae* 1000 Genomes Consortium (2017): Ag1000G phase 2 AR1 data release).

### Identifying UCEs

To identify invariant regions, we used only parts of the multi-species alignment where sequence data were available for all 21 strains. We used Variscan v2.03 (Vilella *et al.* 2005) to find regions of the alignment of 18 bp or longer containing no variation. We mapped the resulting regions back to the PEST reference genome using BWA-aln with strict mapping parameters (zero edit distance, no gap opening allowed; bwa-0.7.10 (Li and Durbin 2010)). Sequences that mapped at multiple places in the genome were included in the analysis but flagged as “repeat sequences” as these would not be suitable for use as CRISPR targets. A recent bioinformatics resource has been published that provides an automated alternative to these methods (Kranjc *et al.* 2021).

We used BEDTools (Quinlan and Hall 2010) to classify the genomic location of the UCEs (such as exonic, intronic, *etc*). The AgamP4.12 base features file was used from VectorBase (Giraldo-Calderón *et al*. 2014). Genic sequences were defined as those with an AGAP gene annotation so include exons, UTRs, and introns. UCEs that partly or wholly fell within genes were classified by us as genic, and those outside genes were classified as intergenic. Results are presented per chromosome arm; *Anopheles* chromosomes contain fixed and polymorphic inversions that can impact evolutionary influences, so treating the autosomes as a single unit would not be appropriate.

For comparison, we used the same method to identify invariant sequences of 18 bp or more just in the *An. gambiae* complex species (*An. gambiae PEST, An. gambiae s.s., An. coluzzii, An. merus, An. arabiensis, An. quadriannulatus*, and *An. melas*). We also looked to see whether the *Anopheles* UCEs were conserved at an older evolutionary scale in *Culex quinquefasciatus* and *Aedes aegypti*. The simplest way to achieve this was to use blastn with default parameters (Altschul *et al.* 1990) in VectorBase to search for similar sequences in the *Aedes* and *Culex* reference genomes (AaegL5.0 and CulPip1.0). Because many of our UCEs were short (18 bp) and may have random hits in the similarity search, we extended the sequences with 50 bp in either side from the *An. gambiae* PEST reference genome. The similarity results from blastn were filtered manually to extract DNA sequences of 18 bp or more that were completely invariant, *i.e.*, included no substitutions or indels, within the *Anopheles* UCE sequences.

### Random control sequences

So that we could compare the location of UCEs with non-UCEs, we used custom Python scripts to extract 10 independent randomly distributed sets of control sequences from the MAF (only from locations where aligned sequences for all 21 species were present) that were matched to give the same number of sequences with the same base-lengths. To compare variation in the Ag1000G data in UCEs and non-UCEs, we also extracted 10 independent sets of control sequences from the AgamP4 genome but also matching for genic and intergenic locations. The custom scripts can be found on GitHub (https://github.com/soloughlin-hub?tab=repositories).

### Orthology between species

For UCEs that fell within genes, we compared the orthology identifiers between AgamP4 and *An. arabiensis* Dongola reference genomes, and between *An. gambiae* PEST and *An. funestus* FUMOZ reference genomes. We chose these species because *An. gambiae* (and its sister species *An. coluzzii*), *An. arabiensis*, and *An. funestus* are the most important malaria vectors in sub-Saharan Africa. *An. gambiae* PEST is a hybrid strain of *An. gambiae* and *An. coluzzii* (previously known as S and M forms of *An. gambiae*). *An. gambiae* and *An. arabiensis* are closely related (in the same species complex) and *An. funestus* is more distantly related. Genic UCEs were checked for orthology between *An. gambiae* and *An. arabiensis* and between *An. gambiae* and *An. funestus*. Coordinates of UCEs were extracted from the multiple-alignment file for *An. arabiensis* and *An. funestus* reference genomes, and annotated with gene names from the base features files *Anopheles*-arabiensis-Dongola_BASEFEATURES_AaraD1and *Anopheles*-funestus-FUMOZ_BASEFEATURES_AfunF1.3 (from VectorBase). Orthology identifiers for each gene in each species were found from the ODBMOZ2_Anophelinae database at OrthoDb.org (Kriventseva *et al.* 2019). Orthology identifiers that match between species indicated that the genes were orthologous. We could not use orthology to directly compare intergenic UCEs, so instead, we identified flanking genes for each intergenic UCE in the reference genome of each species and then compared the orthology identifiers for these genes as before.

### Ontology analysis of genes containing UCEs

PANTHER software (version 14.0) (Mi *et al.* 2017) was used to categorize the gene ontology (GO-Slim) terms of the genes containing UCEs. A gene was represented in the analysis once, regardless of how many UCEs it contained. We performed functional classification by GO-Slim molecular function, biological process, and cellular component terms.

Because the Panther functional classification tool does not take into account how much of the genome is covered by each GO term, we used GOseq (Young *et al.* 2010) to carry out length-bias corrected gene ontology (GO) enrichment analysis, implemented in Galaxy (Afgan *et al.* 2018). GOseq corrects for gene length using a Wallenius non-central hypergeometric distribution. We used GO-Slim terms extracted from VectorBase (Giraldo-Calderón *et al.* 2014) for AgamP4.12 gene set. GO terms with a Benjamini-Hochberg corrected false discovery rate (FDR) of ≤0.05 were considered over-represented. We also looked for over-representation of GO-Slim terms in the genes flanking intergenic UCEs. We were interested to see how our set of UCEs compared with UCEs from *Drosophila* studies, so as well as our full data set, we also performed the GO term analysis on a subset of genes that contained at least one UCE over 50 bp long, to make the data comparable.

### Targets for mosquito control

One form of gene drive aimed at population suppression looks to disrupt essential mosquito genes and thereby impose a strong reproductive load on the population as it spreads. UCEs may offer good targets for control of *An. gambiae* by a gene drive method; if any sequence variation at these sites results in high fitness costs, there would be little selective advantage to a mosquito having the variant allele over the gene drive allele. We searched the functional annotations of genes containing UCEs to find genes that may have a suitable function to be targeted for control. Gene descriptions were obtained from VectorBase (Giraldo-Calderón *et al.* 2014). Gene drives that confer recessive female sterility are particularly potent since both sexes can transmit the drive at very high rates to offspring yet only females homozygous for the drive display the phenotype, which results in a drastic reduction of the population’s reproductive capacity (Burt 2003, Burt and Deredec, 2018). P-sterile values were available for some genes (Hammond *et al.* 2016). P-sterile is a sterility index based on a logistic regression model that correlates gene expression features in *Anopheles* with the likelihood that mutations of the gene produce female sterile alleles in the model dipteran *Drosophila melanogaster* (Baker *et al.* 2011).

To narrow down the gene list to potential vector control targets, we leveraged a large amount of phenotype data already available for *Drosophila* mutants. Where possible, *Drosophila* orthologues were identified for genes containing UCEs (in Vectorbase). We used an ID converter in FlyBase (Gramates *et al.* 2017) to batch convert *Drosophila* gene identifiers into alleles associated with the genes (FBal numbers). The alleles have associated phenotype data provided by the research community; we searched for phenotypes conferring female sterility or recessive lethality.

### Transcription factor binding site motifs in UCEs

We used the “Find Individual Motif Occurrences” (FIMO, Grant *et al.* 2011) scanning module (MEME suite 4.12.0, Bailey *et al.* 2009) to look for transcription factor binding motifs in UCEs and controls. The UCEs were scanned for known insect transcription factor binding sites using weighted matrices from the JASPER CORE collection (Insect position frequency matrices 8th release (2020), Khan *et al*. 2017). The results were filtered by q-value to account for multiple tests. A cut-off of *q* < 0.05 was used.

### Variation at UCE locations in Ag1000G data

Using the final filtered variant file from phase 2 of the Ag1000G project (The *Anopheles gambiae* 1000 Genomes Consortium (2017): Ag1000G phase 2 AR1 data release) we extracted single nucleotide polymorphisms for the UCEs identified above, and for matched non-UCE regions. Diversity statistics were calculated in scikit-allel v1.3.2 (Miles *et al.* 2020): number of segregating sites (s), nucleotide diversity (pi), and the neutrality test Tajima’s D (Tajima 1989).

### Data availability

Data used in this study are publicly available from the *Anopheles* 16 genomes consortium and the *Anopheles gambiae* 1000 Genomes project. Data generated in this study are given in the Supplementary Tables S2 and S3, deposited along with Supplementary Table S1 and Supplementary figures are available at figshare. Custom scripts used in the data analysis can be found at https://github.com/soloughlin-hub?tab=repositories.

Supplementary material is available at https://doi.org/10.25387/g3.14179985.

## Results

### Ultra-conserved regions from the multi-species alignment

Much of the MAF file does not include alignments of all 21 species and strains (Table S8 in Neafsey *et al.* 2015). The total number of aligned bases from which we extracted the UCEs was 17,095,206 (7.4%) of the AgamP4 reference genome (Supplementary Table S1). A total of 8338 invariant regions of 18 bp or more were identified; 1675 on chromosome arm 2 L, 3015 on chromosome arm 2 R, 1375 on chromosome arm 3 L, 2188 on chromosome arm 3 R, and 85 on chromosome X (Table 1; we have also included the same metrics at different evolutionary timescales for comparison). The longest UCE was 164 bp. Genomic coordinates of the UCEs relative to the *Anopheles gambiae* PEST reference genome are given in Supplementary Table S2. The UCEs were distributed throughout the chromosomes, but were under-represented on the X chromosome (0.24% of MAF compared with 1.38% in autosomes; Supplementary Figure S1 and Table S1). The X chromosome is already under-represented in the MAF as it was less alignable than other chromosomes (Figure 2 in Neafsey *et al.* 2015). It is well established that the X chromosome shows higher differentiation between species than autosomes (due to “Haldanes Rule” and the “Large X effect”) and genomic studies have reinforced this observation (Presgraves 2018). However, the under-representation in the MAF is not sufficient to explain the paucity of UCEs on the X. In the *Anopheles* genus, the X chromosome was observed to have undergone particularly dynamic evolution, with chromosome rearrangements at a rate of 2.7 times higher than the autosomes, and a significant degree of observed gene movement from X to other chromosomes relative to *Drosophila* (Neafsey *et al.* 2015). This dynamic evolution of the chromosome may explain why it would be less likely to contain functional sequences that require conservation at the nucleotide level.

**Table 1:** Number of ultra-conserved sequences of 18 bp or more, and total number of invariant sites within these sequences

	2L	2R	3L	3R	X
*Gambiae* complex					
No. UCEs	452,281	612,824	376,383	498,473	99,561
No. Invariant bases within UCEs	15,365,491	21,350,270	12,886,437	17,278,830	3,338,454
*Anopheles*					
No. UCEs	1,675	3,015	1,375	2,188	85
No. Invariant bases within UCEs	45,916	81,186	37,102	59,055	2,299
*Anopheles+Aedes*					
No. UCEs	278	344	193	293	15
No. Invariant bases within UCEs	8,161	10,275	5,499	8,339	456
*Anopheles+Culex*					
No. UCEs	279	350	202	310	16
No. invariant bases within UCEs	8,201	10,184	5,716	8,691	503
*Anopheles+Aedes+Culex*					
No. UCEs	192	247	133	217	12
No. invariant bases within UCEs	5,995	7,579	3,989	6,391	393

Numbers are displayed per chromosome arm, relative to AgamP4 reference genome. *Gambiae* complex, 7 species and strains (*An. gambiae PEST, An. gambiae s.s*. *An. coluzzii, An. merus, An. arabiensis, An. quadriannulatus, An. melas*); *Anopheles*, 21 species and strains; *Culex*, *Culex quinquefasciatus* reference genome; *Aedes*, *Aedes aegypti* reference genome.

Size distributions of the UCEs are shown in Supplementary Figure S2. In the autosomal genic UCEs, there is a pattern of a jump in frequency every three bases, indicating the tendency for runs of ultra-conserved bases to neither start nor end on third codon positions in coding regions. As has been seen in some previous studies (*e.g.*Walter *et al.* 2005; Chiang *et al.* 2008), UCEs are significantly more AT-rich than random control sequences (64% and 54%, respectively, *t*-test *P* < 0.001).

**Figure 1:**
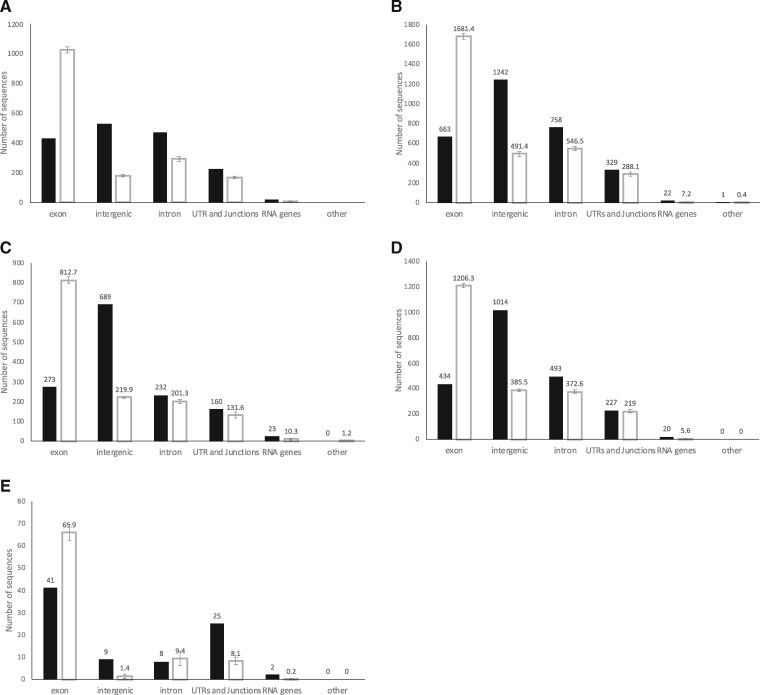
Distribution of UCE and non-UCE control sequences according to genomic location. Genomic locations annotated with BEDtools. Black bars: UCEs; Clear bars: Control sequences. Control error bars: standard deviation for 10 control data sets of sequences of matched length and number to the UCEs, extracted randomly from the MAF, only from regions where sequence for all 21 genomes is present.

We annotated the UCEs in BEDtools to identify where they were found in the genome with regards to exons, introns, UTRs, intergenic regions, *etc* (Figure 1). The 21-genome aligned parts of the MAF file from which we extracted the UCEs is not a representative of the reference genome with respect to these features, so we extracted randomly distributed sets of “control” sequences from the MAF, and only from sequences where all 21 genomes were aligned. These control sequences were matched to give the same number of sequences with the same base-lengths as the UCEs, and were compared with the UCE locations to see whether the UCEs were randomly distributed. The UCE sequences were significantly over-represented (compared with control sequences) in intergenic regions (42% *vs* 15%, *t*-test, *P* < 0.05) and in RNA genes (1% *vs* 0.4%, *t*-test, *P* < 0.05), and less frequent in exons (22% *vs* 57%, *t*-test, *P* < 0.05). The MAF itself is heavily skewed toward exonic sequences, as only about 7% of the *An. gambiae* genome as a whole is exonic (Holt *et al*. 2002).

### Orthology between important vector species

**Figure 2:**
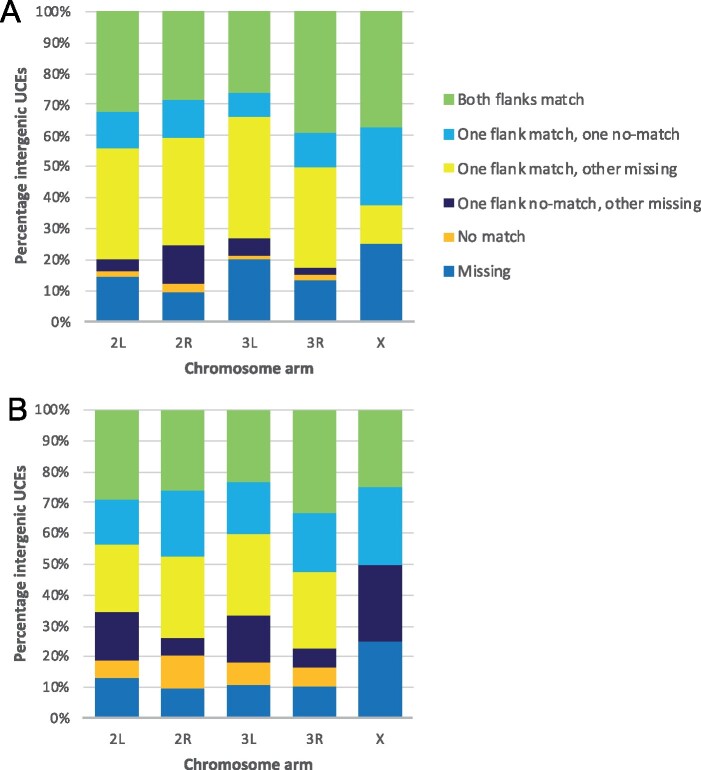
Number of intergenic UCEs that show synteny between (A) *An. gambiae* and *An. arabiensis* and (B) *An. gambiae* and *An. funestus*. The results are shown in six categories: matching orthology of both flanking genes, matching orthology of one flanking gene with no orthology on the other flank, matching orthology of one flanking gene with missing data on the other flank, no orthology on one flank with missing data on the other flank, no orthology of either flanking gene, and missing data on both flanks.

The algorithm that was used to create the sequence alignments in the MAF file results in short blocks of sequences, and is agnostic to genomic location, so to ensure that the location of our UCEs is not random, we checked for orthology between some species in the UCEs. For UCEs that fell within genes, this was done simply by comparing orthology identifiers (from OrthDB.org) between *An. gambiae* and *An. arabiensis*, and between *An. gambiae* and *An. funestus*. For *An. gambiae* and *An. arabiensis*, 94% of autosomal genes containing UCEs shared orthology. For *An. gambiae* and *An. funestus*, this number was 87%. The proportion of UCE-containing genes with orthology between species was lower on the X chromosome (54% for *An. gambiae/An. arabiensis* and 63% for *An. gambiae/An. funestus*). For UCEs that were intergenic, we looked at the orthology of the flanking genes. The results fell into six categories: orthology of both flanking genes, orthology of one flanking gene with no orthology on the other flank, orthology of one flanking gene with missing data on the other flank, no orthology on one flank with missing data on the other flank, missing data on both flanks, and no orthology of either flanking gene. Ignoring missing data, 92% of intergenic UCEs showed full or half orthology between *An. gambiae* and *An. arabiensis*, and 77% of UCEs showed full or half orthology between *An. gambiae* and *An. funestus* (Figure 2). Matching orthology implies that the location of the UCEs is the same in each species with regards to shared synteny blocks.

### Functional profile analysis of the genes containing UCEs via GO-term enrichment

Of the 13,796 genes annotated in the *Anopheles gambiae* PEST gene set Agam4.12, 1601 (12.9%) had at least one UCE. We performed functional classification of the genes based on GO-Slim terms for molecular function, biological process, and cellular component (Supplementary Figure S3).

**Figure 3:**
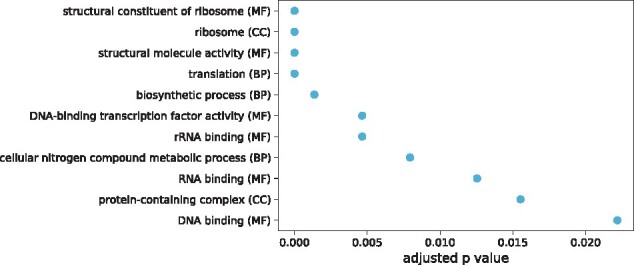
GOseq GO-term enrichment analysis with length-bias correction. GO-Slim categories were extracted from the AgamP4.12 gene set. Results are shown for categories that were enriched with an FDR adjusted *P* < 0.05. MF, molecular function; BP, biological process; CC, cellular component.

Because the functional classification tool does not take into account, the amount of the genome covered by each GO class, we carried out length-bias corrected GO-term enrichment analysis. This showed that certain functional groups were over-represented compared with the whole *Anopheles* PEST reference gene set (Figure 3).

In the genes containing UCEs over 50 bp long, only four categories were over-represented: transmembrane transporter activity (MF), transmembrane transport (BP), transport (BP), and protein-containing complex (CC) (adjusted *P* values 0.0047, 0.0047, 0.0272, and 0.0272, respectively). Genes flanking intergenic UCEs were enriched for the GO-Slim categories DNA binding (MF), DNA-binding transcription factor activity (MF), and anatomical structure development (BP) (adjusted *P* values 4.16E-06, 1.46E-05, and 0.016, respectively).

### Potential targets for vector control

AGAP001189 (odorant-binding protein 10) contained the highest number of invariant bases in UCEs (1215 of 135,306). Nine genes contained UCEs longer than 100 bp, of which three are annotated as being involved in ion transport. These include the voltage-gated sodium channel gene (VGSC, AGAP004707), which is a target for (and therefore has a significant role in conferring resistance to) some of the main classes of insecticides used for malaria vector control. VGSC is one of the most conserved genes we found, containing 13 UCEs with a total of 507 invariant bases, of which 91% were in exons and most coded for trans-membrane domains. A total of 357 genes contained 100 or more invariant bases. A full list of genes containing UCEs is given in Supplementary Table S3.

Eleven genes containing UCEs had a p-sterile score of greater than 0.5 implying that they could be good targets to affect female fertility.

*Drosophila* orthologues were identified for 1309 of the 1601 genes containing UCEs. Allele and phenotype classes for these genes were extracted from Flybase where available. For an effective population suppression gene-drive, the target would affect female fertility or impose a genetic load as a homozygote, so we extracted UCE containing genes that have *Drosophila* orthologues annotated with a female sterile term or a lethal recessive term (shown in Supplementary Table S3). In total, 177 genes containing UCEs have *Drosophila* orthologues with an allele phenotype affecting female fertility, and 367 genes have *Drosophila* orthologues with an allele conferring a lethal recessive phenotype.

### Transcription factor binding motifs in UCEs

**Figure 4:**
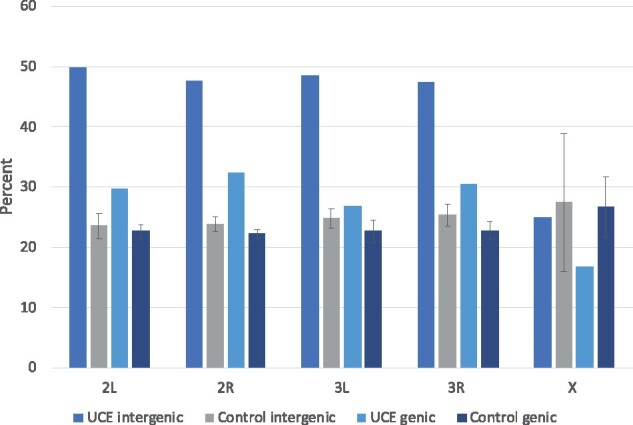
Percentage of UCEs and control sequences that contain at least one insect transcription factor binding motif. Control error bars: standard deviation for 10 control data sets. UCEs were searched for known insect transcription factor binding sites from the JASPER CORE collection (Insect position frequency matrices 8th release (2020), Khan *et al.* 2017). The results were filtered by *q*-value to account for multiple tests. A cut-off of *q* < 0.05 was used.

DNA binding motifs recognized by transcription factors might be expected to be constrained and hence enriched for UCEs since this protein: DNA interaction is sequence-specific. The FIMO search found that 38% of UCEs contained hits for insect transcription factor binding sites with a *q*<0.05 (48% of intergenic and 30% of genic UCEs). For intergenic UCEs, this was significantly higher than control (non-conserved sequences) (48% in UCEs compared with 24% for control sequences of the same number and length, *t*-test across chromosome arms, *P* < 0.005). Within genes, the difference between UCEs and controls was not significant (30% *vs* 23%, *t*-test across chromosome arms ns). This trend did not hold true for the X chromosome, where data are sparse (only 8 intergenic and 75 genic UCEs). Figure 4 shows the percentage of UCEs and control sequences containing transcription factor binding motifs broken down by chromosome arm.

### Genetic variation at UCE locations in Ag1000G data

**Figure 5:**
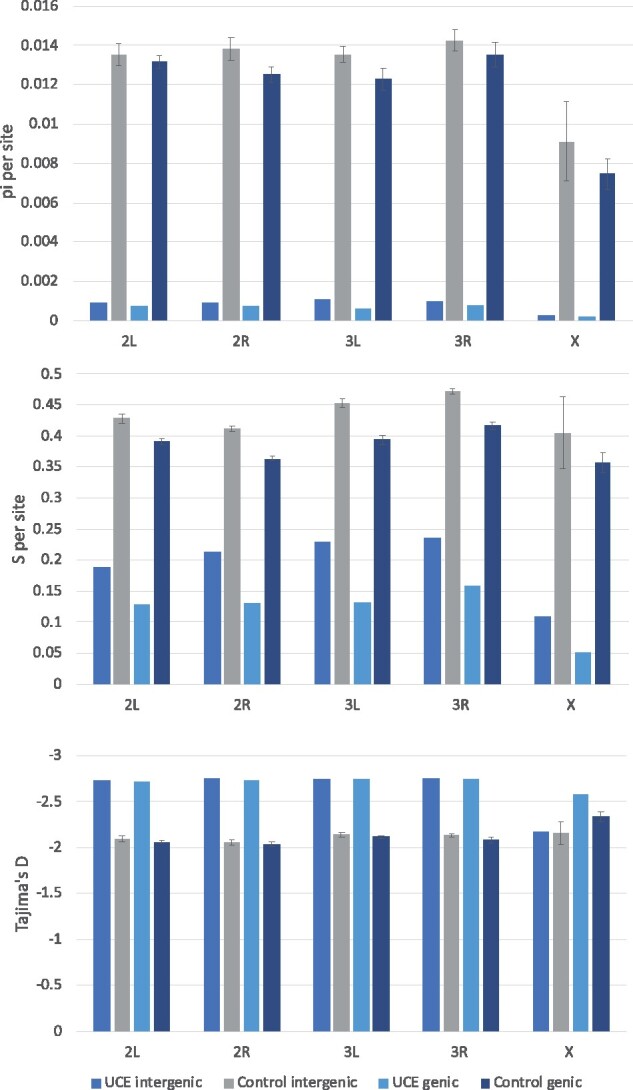
Genetic diversity per chromosome arm in 1,142 *Anopheles gambiae s.l.* samples in UCE locations. Top: nucleotide diversity (π); middle: segregating sites (s); bottom: Tajima’s D. Calculations were made in scikit-allel v1.3.2 (Miles *et al.* 2020). Results are shown per chromosome arm, divided into genic (within an annotated AGAP-) and intergenic regions. Control sequences were extracted randomly from the AgamP4 reference genome and matched to UCE sequences for length, number, and genic or intergenic location. Control error bars: standard deviation for 10 control data sets.

To see whether sequences are ultra-conserved across the *Anopheles* genus show variation in wild mosquito populations, we searched for single nucleotide polymorphisms (SNPs) in the 1142 samples from phase 2 of the Ag1000G project. Significance was compared between UCEs and control sequences using a *t*-test across all chromosomes. There were significantly fewer sites containing polymorphisms in UCEs than control sequences (*P* < 0.0001, Figure 5, middle), and those SNPs that were present were at a significantly lower frequency (*P* < 0.0001, Figure 5, top). Of the 8338 UCEs, 1213 (15%) contained no SNPs in the 1142 samples (229 on 2L, 470 on 2R, 226 on 3L, 259 on 3R, and 29 on X). Tajima’s D is significantly different and more negative for UCEs than controls, with the exception of X chromosome intergenic sequences (*P* < 0.005, Figure 5, bottom). Negative values of Tajima’s D are expected for sequences under purifying selection.

The Ag1000G study (*Anopheles gambiae* 1000 genomes consortium *et al.* 2017) performed a search within the Phase 1 data to look for potential Cas9 targets (non-overlapping exonic invariant sequences of 21 bp, ending in the “NGG” motif) within *An. gambiae* and *An. coluzzii*. They identified 13 genes containing sequences matching these criteria. However, none of these sequences corresponded to UCEs fitting our more stringent definition of being conserved across the wider *Anopheles* genus. We did not confine our search for UCEs to current Cas9 target site restrictions because of the growing possibility of relaxation of these constraints as the ability to re-engineer Cas9 tolerance progresses (Walton *et al.* 2020). However, for completeness, we looked within our final set of UCEs for the Cas9 motif (18 bp followed by -NGG, or CCN- followed by 18 bp). We found 1997 (24%) UCEs contained suitable targets for Cas9.

## Discussion

### Similarities and differences of *Anopheles* UCEs with UCEs from *Drosophila*

Despite approximately 100 million years since their most recent common ancestor, we identified in the *Anopheles* genus over 8000 sequences of 18 bp or more where there was no nucleotide variation across the alignment of 21 species and strains. By coincidence, this is approximately the same span of evolutionary time covered in the human/mouse/rat data set in which UCEs were originally identified (Bejerano *et al.* 2004). Approximately 481 UCEs of more than 200 bp were observed between these genomes, but the longest we found in the *Anopheles* genus was 164 bp. This is consistent with previous reports that UCEs are fewer and shorter in insects (mainly *Drosophila*) than vertebrates (Glazov *et al.* 2005; Makunin *et al.* 2013). Our criteria for identifying UCEs were somewhat different than those used previously. First, we only considered sequences that were present in all 21 species/strains in the alignment; some of these species have poorly assembled genomes, so this may have reduced the number of UCEs that we uncovered. Second, we also included invariant stretches of 18 bp or more, whereas *Drosophila* studies have used cut-offs of 50 bp (Glazov *et al.* 2005, Warnefors *et al.* 2016), 80 bp (Kern *et al.* 2015) or 100 bp (Makunin *et al.* 2013). Despite this, we see some similarities between our UCEs and UCEs found in *Drosophila*. UCEs are located in all parts of the genome and, like *Drosophila*, the majority are found in intergenic regions and introns. We also found that junction locations (*e.g.* intron-exon, exon-intergenic, *etc*) are over-represented compared with random sequences, which in *Drosophila* has been linked to the conservation of splice-sites (Glazov *et al.* 2005; Warnefors *et al.* 2016). Another similarity with *Drosophila* is the high proportion of genes with the GO terms “binding” and “transporter activity” (Glazov *et al.* 2005; Kern *et al.* 2015). In *Drosophila*, ion channel/transporter genes have been shown to undergo extensive RNA editing (Hanrahan *et al.* 2000; Hoopengardner *et al.* 2003; Rodriguez *et al.* 2012) which is thought to explain the high level of conservation. This is because RNA adenosine deaminases require double-stranded RNA as a substrate, which means that there is likely to be strong selection at the nucleotide level. The high number of UCEs in *Anopheles* ion channel/transporter genes suggests that a similar mechanism is responsible for the high conservation in the *Anopheles* genus. However, these genes are extremely long and are not over-represented in the UCE data when a length-bias corrected analysis is carried out in GOseq. In the GOseq analysis, the most over-represented molecular functions are mostly involved in binding or structure. Transcription factor binding, enzyme binding, and nucleic acid binding have also been shown to be associated with ultra-conservation in both invertebrates and mammals (Bejerano *et al.* 2004; Glazov *et al.* 2005). A noteworthy addition to highly represented GO terms in *Anopheles* that has not been reported in *Drosophila*, is the category of “catalytic activity” genes, although again, these were not over-represented when gene length was taken into account. When the GO term functional classification was carried out on genes containing UCEs of 50 bp or more in length, we found that the category reduced from 28% to 18% suggesting that these shorter ultra-conserved regions most likely code for a small number of key residues around an active site.

The high number of UCEs that we observe in intergenic regions and introns suggests that we have found numerous unannotated locations in the *Anopheles* PEST reference genome with putative regulatory functions. At least 70% were syntenic between *An. gambiae*/*An. arabiensis* and *An. gambiae*/*An. funestus*, so the location of these highly conserved sequences is likely to be important. A GOseq analysis of the genes flanking these intergenic sequences showed significant over-representation of genes with DNA-binding GO terms (data not shown). Sequences that are ultra-conserved at the nucleotide level across a long evolutionary time have been shown to be linked to regulatory functions such as cis-regulation of genes (*e.g.* enhancers, insulators, silencers) and RNA genes (*e.g.* miRNA and snRNA), likely because of the sequence-specific nature of protein:nucleotide or nucleotide:nucleotide interactions. Of the 77 miRNA genes that are annotated in the *Anopheles* PEST genome, 19 were included in our set of UCEs (other miRNAs may contain ultra-conserved regions that did not meet our criteria). We also found known insect transcription binding factors in 48% of the intergenic UCEs.

### Polymorphisms in UCEs in *Anopheles* populations

All of the UCEs discovered from the alignment of the reference genomes of 21 *Anopheles* species were also found to be highly conserved in the sample of 1142 wild-caught mosquitoes sequenced in phase 1 of Ag1000G. Although the majority of UCEs contained one or more polymorphisms, they were almost all rare. 1213 UCEs showed no polymorphisms at all in this sample. This does not rule out the existence of polymorphisms in the wild populations but does imply that there may be strong constraint at a nucleotide level that means an alteration of the sequence either naturally or by the action of a gene drive may have a strong fitness cost. This would need to be tested experimentally as different levels of underlying functional constraint may have different fitness costs. For example, deletion of certain ultra-conserved sequences in mice gave no discernible fitness cost (Ahituv *et al.* 2007), but a similar experiment in *Drosophila* showed promise, with 4 out of 11 UCEs with inserted transposons having a lethal recessive phenotype (Makunin *et al.* 2013). For a resistance-proof gene drive, selecting target sites that show high levels of conservation is a good starting point, but the targets would need to be tested under selection pressure to ensure that functional mutants do not arise.

### UCEs and vector control

UCEs occur within many genes that could have the potential for vector control. Nearly 200 genes have *Drosophila* orthologues with an allele phenotype affecting female fertility, and over three hundred genes have *Drosophila* orthologues with an allele conferring a lethal recessive phenotype. These phenotypes could both be used for a population suppression strategy, *i.e.*, to reduce the numbers of mosquitoes to a level where malaria could no longer be transmitted (Deredec *et al.* 2011). More investigation would be needed to see whether disrupting the genes at the ultra-conserved loci gives the same phenotype in *Anopheles*. There are also genes that confer recessive phenotypes in *Drosophila* such as “flightless” or “behaviour defective” that could also be used for population suppression, or for a population modification type of strategy, where instead of reducing the mosquito population it is replaced by a strain that cannot transmit malaria (Carballar-Lejarazú and James, 2017). Precise targeting of sequences using CRISPR/Cas9 gene editing had made testing for these phenotypes feasible.

Another potential source of targets for genetic control approaches that has not yet been explored would be to target sequences involved in gene regulation. Many ultra-conserved sequences in mammals and invertebrates are thought to be involved in the regulation of genes important in development (Bejerano *et al.* 2004; Boffelli *et al.* 2004; Sandelin *et al.* 2004; Glazov *et al.* 2005).

Targeting a sequence that is conserved between species means that the gene drive could spread between closely related species that hybridize in the wild. For this to happen, the species would need to mate in the wild, produce some fertile offspring, and be able to express the CRISPR enzyme using the same promoter. Three species (*An. gambiae*, *An. coluzzii*, and *An. arabiensis*) are responsible for the majority of malaria transmission in some parts of sub-Saharan Africa, and are known to hybridize in nature (*e.g.*Weetman *et al.* 2014, Fontaine *et al.* 2015; *Anopheles gambiae* 1000 Genomes Consortium *et al.* 2017). For effective vector control, it would be desirable to be able to reduce or alter all three species with one construct. The gene drive would not spread to *Anopheles* species that do not mate in the wild, so would not spread beyond the *Anopheles gambiae* species complex. If a particular target site was proved to be effective for vector control in *An. gambiae*, a gene drive targeting an orthologous site could be developed in the laboratory for other important malaria vectors such as *An. funestus*.

There may be some circumstances, for example, for phased testing of a gene drive’s efficacy and safety, where it is desirable to target a sequence that is unique to a particular population. For this, it would be interesting to explore conserved sites that show polymorphisms within species, a prospect that is being explored for mosquito and rodent control (Oh *et al.* 2021; Willis and Burt 2021).

### Conclusion

Thousands of short genomic regions exist that are conserved across the *Anopheles* genus. These sequences show many of the same traits as UCEs found in *Drosophila* (such as an association with gene regulation and ion channel activity). Our list of UCEs in the *Anopheles* genus should provide a valuable starting point for the selection and testing of new targets for gene-drive modification in the mosquitoes that transmit malaria. Focusing on sequences that have remained highly conserved over a long evolutionary time has promise for mitigating against or slowing the development of resistant alleles in the wild population.
